# Effect of the strain on spin-valley transport properties in MoS_2_ superlattice

**DOI:** 10.1038/s41598-021-97189-4

**Published:** 2021-09-02

**Authors:** Farhad Sattari, Soghra Mirershadi

**Affiliations:** 1 Department of Physics, Faculty of Sciences, University of Mohaghegh Ardabili, P.O. Box 179, Ardabil, Iran; 2 Nanoscience and Nanotechnology Research Group, University of Mohaghegh Ardabili, Ardabil, Iran; 3 Department of Engineering Sciences, Faculty of Advanced Technologies, University of Mohaghegh Ardabili, Namin, Iran

**Keywords:** Materials science, Nanoscience and technology

## Abstract

The effect of the strain on the spin and valley dependent transport properties, including the conductance and polarization, through a monolayer MoS_2_ superlattice under Rashba spin–orbit coupling is theoretically investigated. It is found that the conductance strongly depends on the spin and valley degrees of freedom, and spin-inversion can be achieved by MoS_2_ superlattice. Also, the spin and valley dependent conductance in a monolayer MoS_2_ superlattice can be efficiently adjusted via strain and the number of the superlattice barriers. Moreover, it is demonstrated that both the magnitude and sign of the spin and valley polarization depend on the strain strength, the number of barriers, and electrostatic barrier height. Both full spin and valley polarized current (with 100% or − 100% efficiency) can be realized in a MoS_2_ superlattice under strain.

## Introduction

In recent years, two-dimensional (2D) materials have been attracted extensive interests due to their potential applications in various research fields. Graphene^1,2^ is currently the most important member of the 2D materials family. Since monolayer graphene is a gapless semiconductor, it has no useful applications in the semiconductor industry, logic, and spintronic devices. Within the 2D materials, monolayer molybdenum disulfide (MoS_2_) was successfully synthesized via several experimental techniques^3–7^. Unlike graphene, the monolayer MoS_2_ is a direct bandgap semiconductor with a tunable bandgap^8^. Due to the heavy transition-metal atoms, the monolayer MoS_2_ has a strong spin–orbit coupling (SOC)^9^. Furthermore, in the monolayer MoS_2_, in the Brillouin zone two inequivalent valleys (K and K′) are separated by a large momentum^10^. Additionally, due to the symmetry breaking and large SOC in the monolayer MoS_2_, it is possible to control and tune the spin and valley polarization properties in the monolayer MoS_2_-based systems^11–13^. Moreover, the monolayer MoS_2_, in the presence of the Rashba spin–orbit coupling (RSOC), is a fascinating material for spintronics applications. In the monolayer MoS_2_ the RSOC can easily be induced and tuned via an external electric field^14^ or a ferromagnetic exchange field^15^. On the other hand, the electronic, optical, and transport properties of the monolayer MoS_2_ can be modulated by applying an external strain^16–20^. The strain can be induced in the MoS_2_ sheet by substrate^21^ or during the CVD growth^22^. In recent years, electron, spin and valley-dependent transport properties were reported extensively in monolayer MoS_2_ structures, both experimentally and theoretically^23–36^. Fontana et al.^23^ experimentally investigated the transport properties of the electron and hole in a gated MoS_2_ Schottky barrier and found that in this structure, the source and drain electrodes’ materials are essential keys in controlling the transport through the conduction or valence band. Rotjanapittayakul et al.^27^ theoretically studied the magnetoresistance and spin injection in a MoS_2_ junction, and demonstrated that a magnetoresistance and spin injection efficiency of the order of 300% and 80%, respectively, can be observed in a MoS_2_-based tunnel junction. The effect of the Rashba spin–orbit interaction on the thermoelectric properties of monolayer MoS_2_ nanoribbon is described in Ref.^31^, in which the authors presented that the magnitude and sign of Seebeck thermopower can be tuned by adjusting the structure's parameters. Besides, the superlattices structures^37^ provide a new way for controlling the transport properties. Recently a great deal of attention has been addressed to the transport properties in superlattice-based 2D materials^38–50^. Yu and Liu^41^ studied the spin transport properties through monolayer and bilayer graphene superlattice. They showed that the monolayer and bilayer graphene superlattice with zigzag boundaries could be used for perfect spin-filtering. Zhang et al.^42^ demonstrated that a controllable spin and valley polarized current could be obtained in a silicene superlattice in the presence of the electric and magnetic field. The effect of the strain on the electronic properties in the MoS_2_-WSe_2_ moiré superlattice was investigated by Waters et al.^49^. They found that in-plane strain and out-of-plane deformations significantly impact the MoS_2_-WSe_2_ moiré superlattice's electronic properties. In the present paper, we propose a monolayer MoS_2_ superlattice in the presence of strain and RSOC in order to achieve a full spin and valley polarized current. We demonstrate that the spin and valley polarization's magnitude and sign depend on the strain strength and the superlattice parameters. The results show that the conductance dependent on the spin and valley degrees of freedom and spin-inversion can be obtained by the MoS_2_ superlattice.

## Model and methods

**Figure 1:**
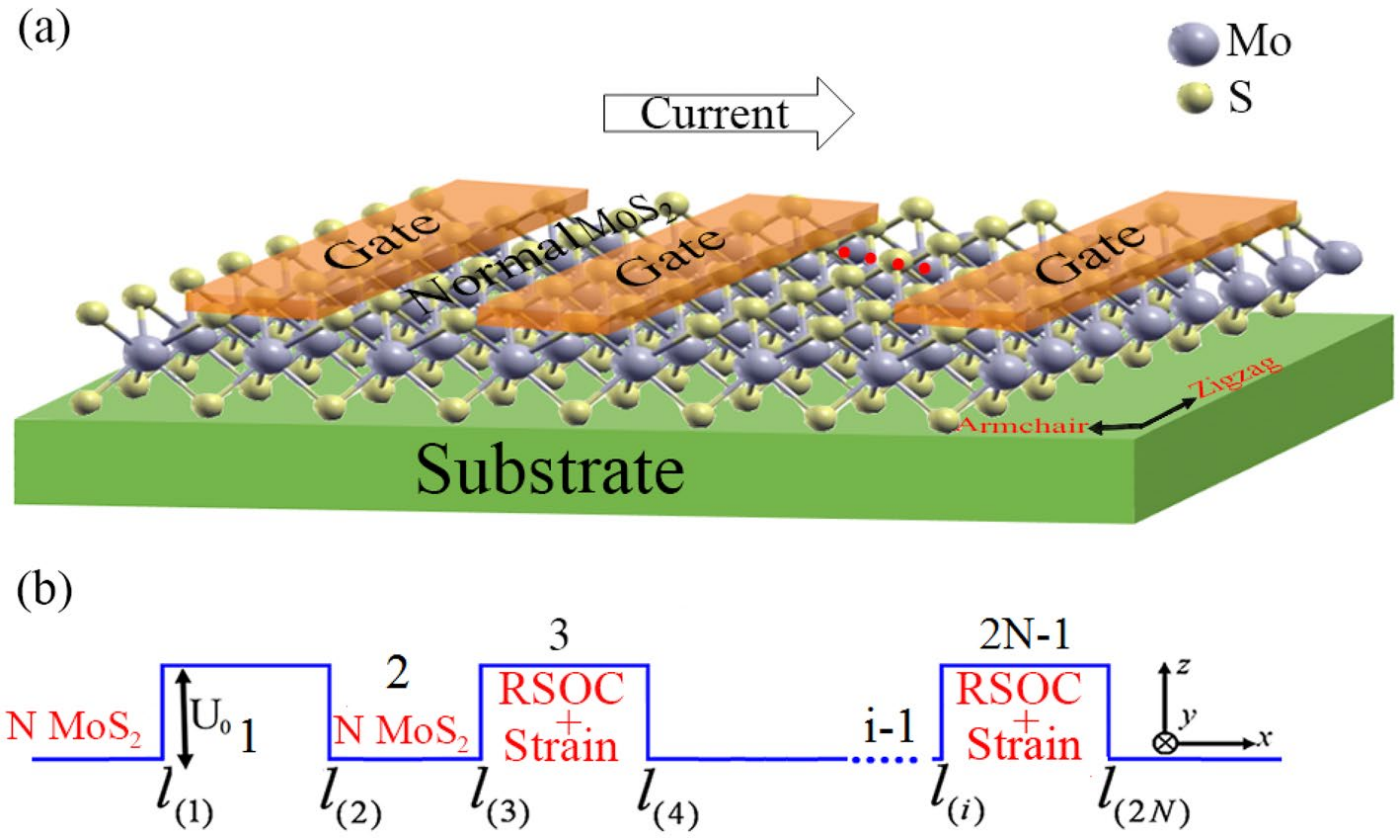
(**a**) Schematic representation of a monolayer MoS_2_ superlattic under strain and RSOC. (**b**) Energy potential profile of MoS_2_ superlattic with* N* electrostatic potential barrier.

In this work, we are interested in the spin- and valley-dependent transport properties in a MoS_2_ superlattice with RSOC in the presence of a uniaxial strain. A series of metallic gate voltages on the top of monolayer MoS_2_ with a suitable substrate can be used to get a monolayer MoS_2_ superlattice in the presence of RSOC and strain. In our model, the RSOC region with a gate voltage and strain (barrier region) is separated by a normal monolayer MoS_2_ (N MoS_2_), in which there is no RSOC and strain (well region). The schematic of our proposed device structure is shown in Fig. 1. The growth direction of the superlattice is along the *x-*axis. We assume that the strain is applied in the armchair direction. The strain tensor, є, can be written as follows^20^

1 $$ \epsilon = \varepsilon \left( {\begin{array}{*{20}c} {\cos^{2} \alpha - \mu \sin^{2} \alpha } & {(1 + \mu )\cos \alpha \sin \alpha } \\ {(1 + \mu )\cos \alpha \sin \alpha } & {\sin^{2} \alpha - \mu \cos^{2} \alpha } \\ \end{array} } \right), $$

where $\alpha$ is the angle between the *x*-axis and the direction of the strain. For the armchair direction strain $\alpha = 0$. $\varepsilon$ is the strain strength and $\mu = 0.25$ is the Poisson’s ratio for the MoS_2_^51^. In the considered structure, the low-energy effective Hamiltonian of the carriers near the K and K′ valleys can be written as:

2 $$ \hat{H} = \hat{H}_{0} + \hat{H}_{RSOC} , $$

with

3 $$ \begin{aligned} & \hat{H}_{0} = \hbar v_{F} U^{\dag } (\alpha )[(\eta (1 - \lambda_{x} \varepsilon )k_{{s^{\prime}(s)}} \sigma_{x} + (1 - \lambda_{y} \varepsilon )q_{y} \sigma_{y} )]U^{\dag } (\alpha ) + (\Delta + \eta s_{z} \lambda )\sigma_{z} + \eta s_{z} \lambda + V(x)\hat{I}, \\ & \hat{H}_{RSOC} = \lambda_{R} (s_{y} \otimes \sigma_{x} - s_{x} \otimes \sigma_{y} ), \\ \end{aligned} $$

4 $$ V(x) = \left\{ {\begin{array}{*{20}c} {U_{0} } & {{\text{in}}\;{\text{barrier}}} \\ 0 & {{\text{in}}\;{\text{well}}} \\ \end{array} } \right. $$

Here, $\hat{\sigma } = (\sigma_{x} ,\sigma_{y} ,\sigma_{z} )$ and $s = (s_{x} ,s_{y} ,s_{z} )$ are the Pauli matrices for the sublattice and the spin spaces, respectively. $v_{F} \approx 5.3 \times 10^{5}$ m/s denotes the Fermi velocity in a monolayer MoS_2_, $\eta = + 1/ - 1$ is the valley index ($\eta = 1$ for K and $\eta = - 1$ for K′ valley) and $s_{z} = + 1( - 1)$ denotes the electron with the spin-up (down). $\Delta = 833$ meV is the energy gap^12^ in the monolayer MoS_2_, $\lambda_{R}$ is RSOC strength and $\lambda = 37.5$ meV is the spin-splitting energy of the valence band caused by the spin–orbit coupling^12,15^. $U_{0}$ displays the electrostatic potential barrier's height, $\hat{I}$ is a unitary matrix, $$U(\alpha ) = diag(1,\begin{array}{*{20}c} {e^{i\alpha } } \\ \end{array})$$ denotes the unitary matrix, which performs a rotation in the sublattice space^52^ and for the monolayer MoS_2_ $\lambda_{x} = 2.2$ and $\lambda_{y} = - \;0.5$^53^. Also, the longitudinal $k_{x}$ ($k_{{s^{\prime}(s)}}$) and the transverse $k_{y}$ ($q_{y}$) components of the wave vectors in the N MoS_2_ regions (in the ERSOC regions) with $k_{{}}$ ($k^{\prime}_{{s^{\prime}(s)}}$), respectively. $k_{{}}$ and $k^{\prime}_{{s^{\prime}(s)}}$ are given by:

5 $$ k = ((\eta k_{x} )^{2} + k_{y}^{2} ) = \frac{1}{{\hbar v_{F} }}\sqrt {(E - 2\eta s_{z} \lambda + \Delta )(E - \Delta )} , $$

6 $$ \begin{gathered} k^{\prime}_{{s^{\prime}(s)}} = ((\eta k_{{s^{\prime}(s)}} )^{2} (1 - \lambda_{x} \varepsilon )^{2} + q_{y}^{2} (1 - \lambda_{y} \varepsilon )^{2} )  \hfill \\ = \sqrt {(E - \Delta - U_{0} )(E + \Delta - U_{0} - 1( - 1)2\eta \lambda - 1( - 1)2\eta \lambda_{R} )/\hbar^{2} v_{F}^{2} } . \hfill \\ \end{gathered} $$

where, $k_{{s^{\prime}(s)}} = (1 - \lambda_{x} \varepsilon )^{ - 1} k^{\prime}_{{s^{\prime}(s)}} \cos \theta$ and $q_{y} = (1 - \lambda_{y} \varepsilon )^{ - 1} k^{\prime}_{{s^{\prime}(s)}} \sin \theta .$ Let us now consider electrons with the angle of incidence of $\varphi$, spin *s* and energy *E* will go towards the monolayer MoS_2_ superlattice from the left side. The spin and valley dependent wave function in the RSOC ($\psi_{s,\eta }^{ \pm }$) and the normal ($\psi_{Ns,\eta }^{ \pm }$) regions can be given by:

7 $$ \begin{aligned} & \psi_{N \uparrow \eta }^{ \pm } = (( \pm \eta k_{x} - ik_{y} ),\begin{array}{*{20}c} {(E - \Delta )/\hbar v_{F} ,0,\begin{array}{*{20}c} 0 \\ \end{array} } \\ \end{array} )e^{{i( \pm k_{x} x + k_{y} y)}} \times A, \\ & \psi_{N \downarrow \eta }^{ \pm } = (0,\begin{array}{*{20}c} 0 \\ \end{array} ,( \pm \eta k_{x} - ik_{y} ),\begin{array}{*{20}c} {(E - \Delta )/\hbar v_{F} } \\ \end{array} )e^{{i( \pm k_{x} x + k_{y} y)}} \times A, \\ & A = 1/\sqrt {2(\left| {k_{x} } \right|^{2} + k_{y}^{2} + ((E - \Delta )/\hbar v_{F} )^{2} )} , \\ \end{aligned} $$

8 $$ \begin{aligned} & \psi_{ \uparrow ( \downarrow )\eta }^{ \pm } = \left\{ {[(( \pm \eta k_{ \uparrow ( \downarrow )} )(1 - \lambda_{x} \varepsilon )) - ((1 - \lambda_{y} \varepsilon )iq_{y} )]} \right.,\begin{array}{*{20}c} {\begin{array}{*{20}c} {[E - \Delta - U_{0} ]/\hbar v_{F} ,\begin{array}{*{20}c} {\begin{array}{*{20}c} { - i \times 1( - 1)[E - \Delta - U_{0} ]/\hbar v_{F} }, \\ \end{array} } \\ \end{array} } \\ \end{array} } \\ \end{array} \\ & \;\;\;\;\;\;\left. { - i \times 1( - 1)( \pm \eta (k_{ \uparrow ( \downarrow )} (1 - \lambda_{x} \varepsilon )) + ((1 - \lambda_{y} \varepsilon )iq_{y} ))} \right\} \times e^{{i( \pm k_{ \uparrow ( \downarrow )} x + k_{y} y)}} D_{{_{ \uparrow ( \downarrow )} }} , \\ & D_{{_{ \uparrow ( \downarrow )} }} = 1/\sqrt {2(\left| {k_{{_{ \uparrow ( \downarrow )} }} } \right|^{2} \times (1 - \lambda_{x} \varepsilon )^{2} ) + (q_{y}^{2} \times (1 - \lambda_{y} \varepsilon )^{2} ) + (E - \Delta - U_{0} )/\hbar v_{F} )^{2} } , \\ & k_{ \uparrow ( \downarrow )} = \sqrt {(E - \Delta - U_{0} )(E + \Delta - U_{0} - 2s_{z} \eta \lambda - 2s_{z} \eta \lambda_{R} )/\hbar^{2} v_{F}^{2} - (q_{y}^{2} \times (1 - \lambda_{y} \varepsilon )^{2} )} . \\ \end{aligned} $$

The spin and valley dependent transmission probability, $T_{{s^{\prime}s\eta }}$, (with the spin $s = \uparrow , \downarrow$ to be transmitted to the spin $s^{\prime} = \uparrow , \downarrow$) through the monolayer MoS_2_ superlattice with *N* electrostatic barriers can obtained by applying the boundary conditions and using the transfer matrix approach^54,55^. Then, the spin and valley dependent conductance of a monolayer MoS_2_ superlattice under strain and RSOC is defined as^56^:

9 $$ G_{{s^{\prime}s\eta }} = G_{0} \int\limits_{ - \pi /2}^{ - \pi /2} {T_{{s^{\prime}s\eta }} (\varphi )\cos (\varphi )d\varphi ,} $$

Finally, the spin and valley polarization can be calculated by^57^:

10 $$ P_{S} = \frac{{G_{{ \uparrow \uparrow K_{1} }} + G_{{ \uparrow \uparrow K_{2} }} + G_{{ \uparrow \downarrow K_{1} }} + G_{{ \uparrow \downarrow K_{2} }} - G_{{ \downarrow \uparrow K_{1} }} - G_{{ \downarrow \uparrow K_{2} }} - G_{{ \downarrow \downarrow K_{1} }} - G_{{ \downarrow \downarrow K_{2} }} }}{{G_{{ \uparrow \uparrow K_{1} }} + G_{{ \uparrow \uparrow K_{2} }} + G_{{ \uparrow \downarrow K_{1} }} + G_{{ \uparrow \downarrow K_{2} }} + G_{{ \downarrow \uparrow K_{1} }} + G_{{ \downarrow \uparrow K_{2} }} + G_{{ \downarrow \downarrow K_{1} }} + G_{{ \downarrow \downarrow K_{2} }} }}, $$

11 $$ P_{V} = \frac{{G_{{ \uparrow \uparrow K_{1} }} + G_{{ \uparrow \downarrow K_{1} }} + G_{{ \downarrow \downarrow K_{1} }} + G_{{ \downarrow \uparrow K_{1} }} - G_{{ \uparrow \uparrow K_{2} }} - G_{{ \uparrow \downarrow K_{2} }} - G_{{ \downarrow \downarrow K_{2} }} - G_{{ \downarrow \uparrow K_{2} }} }}{{G_{{ \uparrow \uparrow K_{1} }} + G_{{ \uparrow \downarrow K_{1} }} + G_{{ \downarrow \downarrow K_{1} }} + G_{{ \downarrow \uparrow K_{1} }} + G_{{ \uparrow \uparrow K_{2} }} + G_{{ \uparrow \downarrow K_{2} }} + G_{{ \downarrow \downarrow K_{2} }} + G_{{ \downarrow \uparrow K_{2} }} }}. $$

## Results and discussion

**Figure 2:**
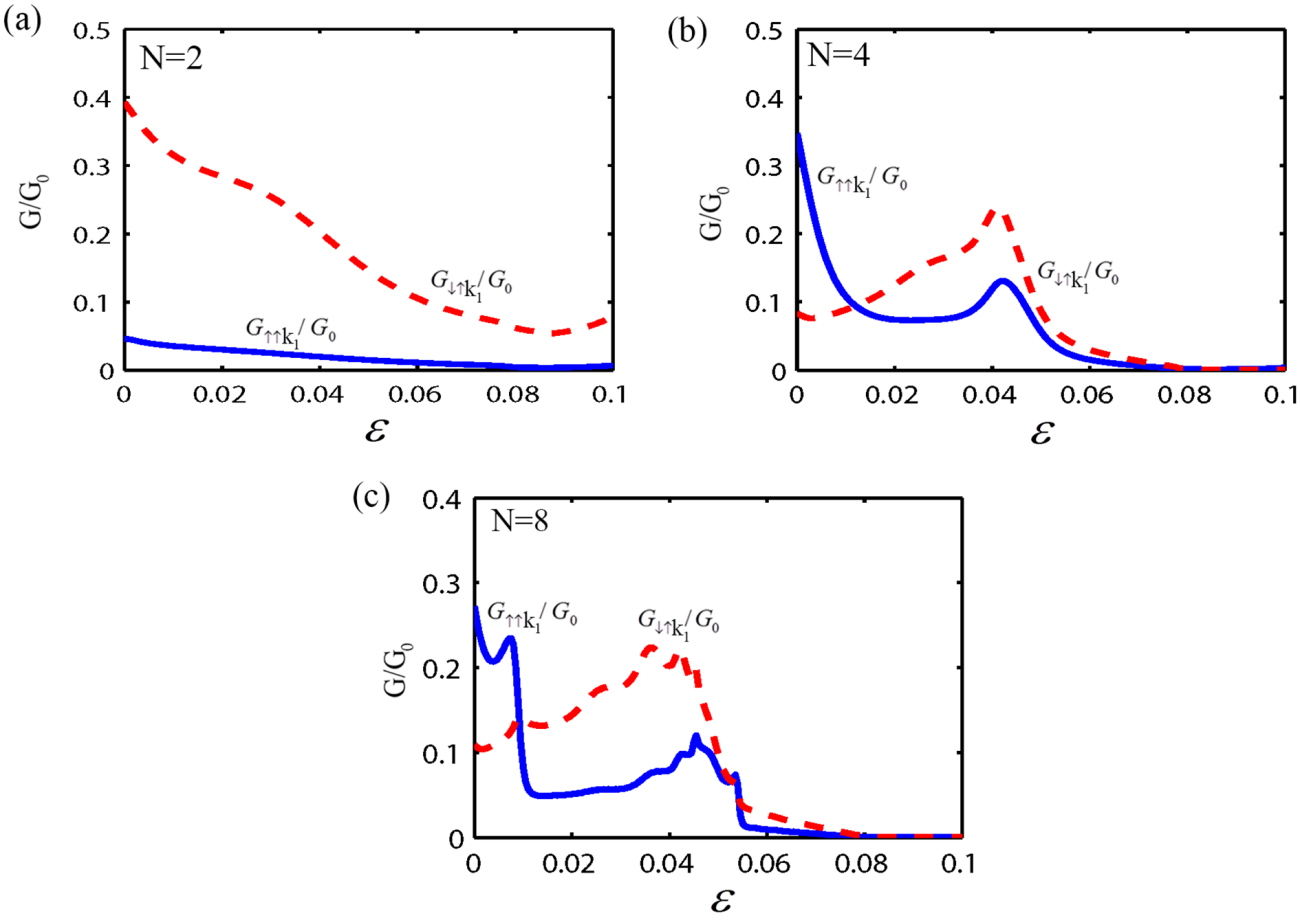
The spin dependent conductance for valley K_1_ as a function of strain strength ($\varepsilon$), for fixed $E = 1.5\Delta$, $U_{0} = 3.5\Delta,$ $\lambda_{R} = 50$ meV, $k_{F0} b = 5$ and $k_{F0} w = 3.$ For (**a**) two barrier, (**b**) four barriers, and (**c**) eight barriers structure.

**Figure 3:**
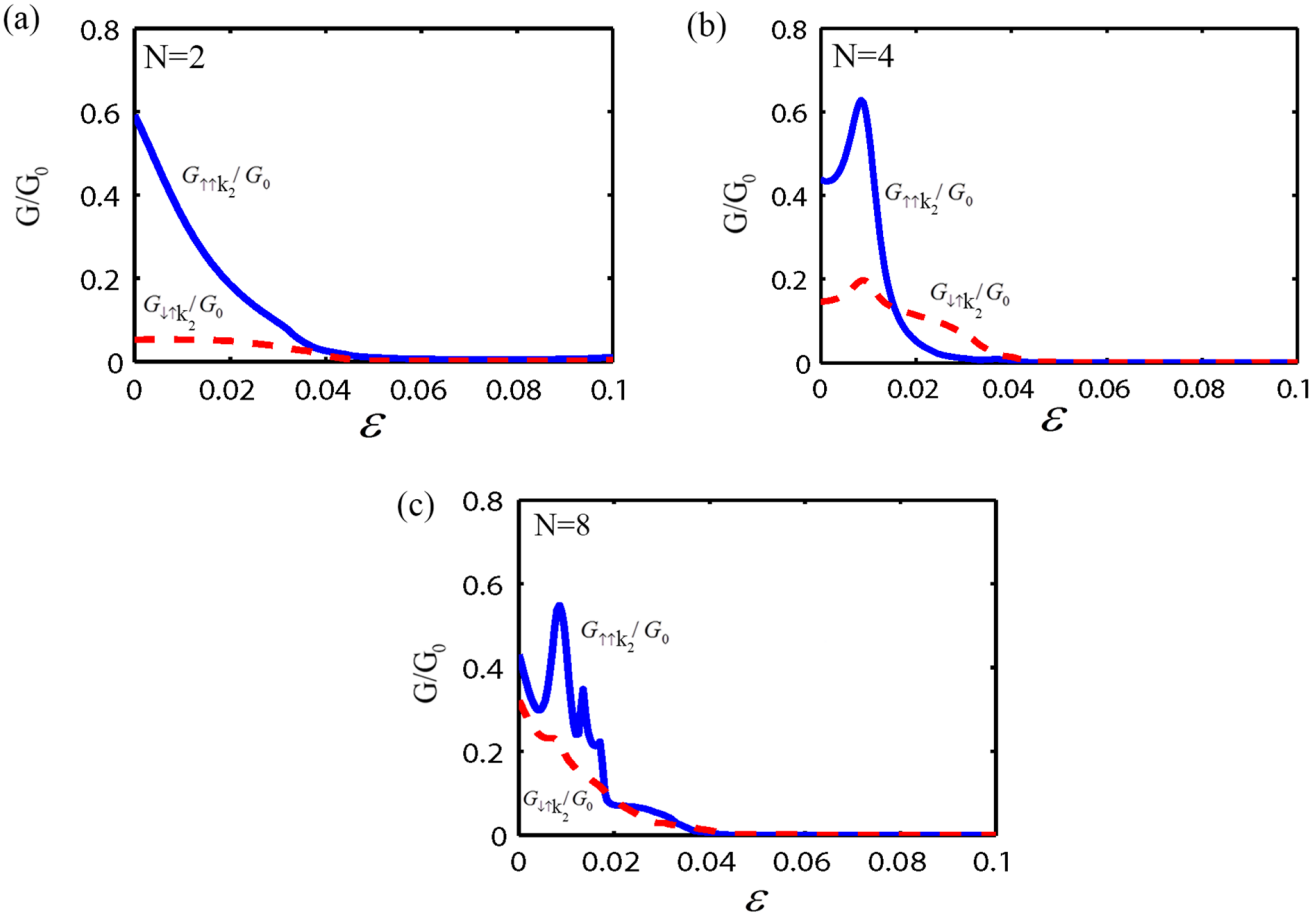
The spin dependent conductance for valley K_2_ versus $\varepsilon$, for fixed $E = 1.5\Delta$, $U_{0} = 3.5\Delta ,$ $\lambda_{R} = 50$ meV, $k_{F0} b = 5$ and $k_{F0} w = 3.$ For (**a**) two barrier, (**b**) four barriers, and (**c**) eight barriers structure.

**Figure 4:**
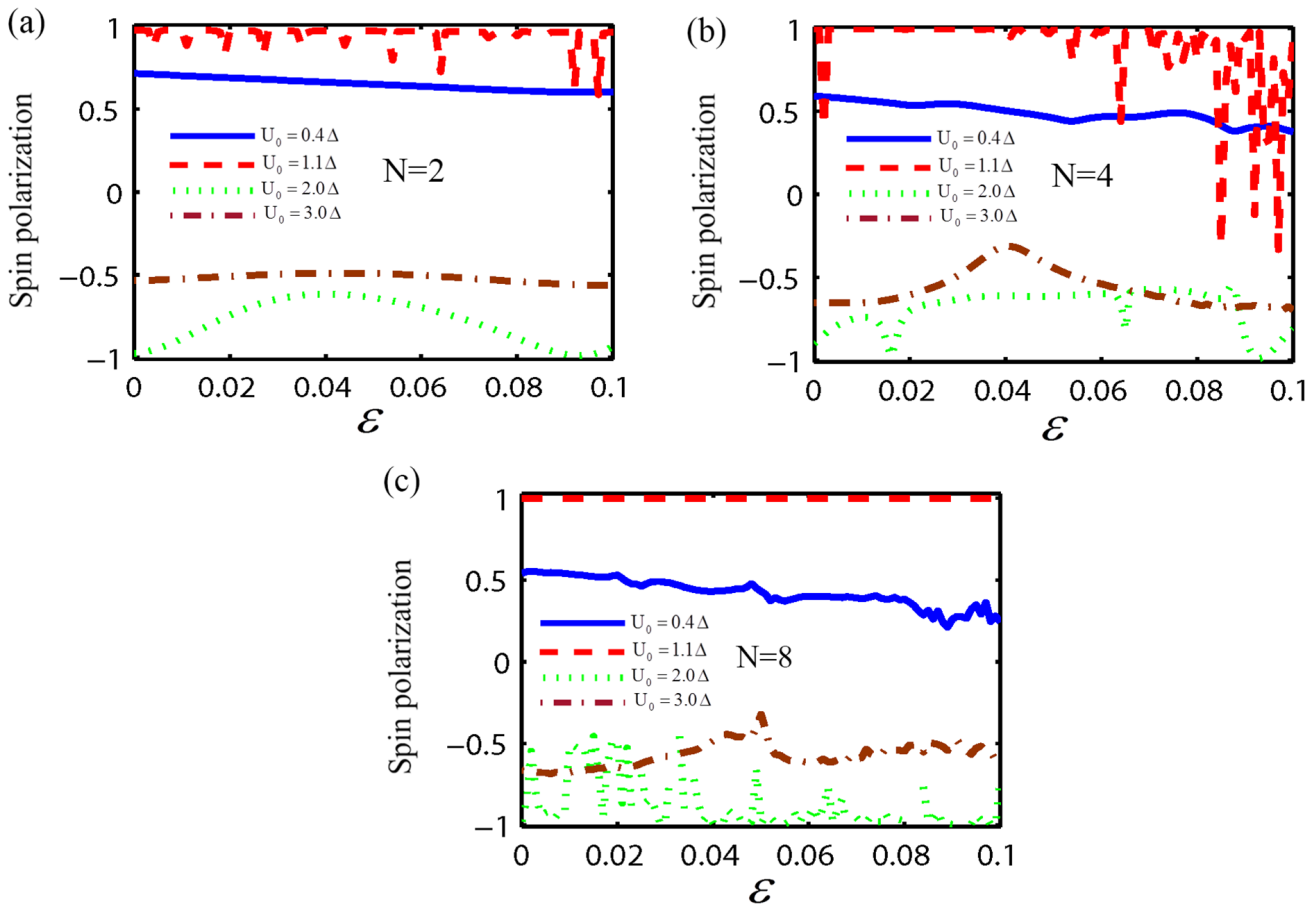
The spin polarization as a function of $\varepsilon$ with several values of the height of the electrostatic potential barrier, for fixed $E = 1.5\Delta$, $\lambda_{R} = 50$ meV, $k_{F0} b = 5$ and $k_{F0} w = 3.$ For (**a**) two barrier, (**b**) four barriers, and (**c**) eight barriers structure.

**Figure 5:**
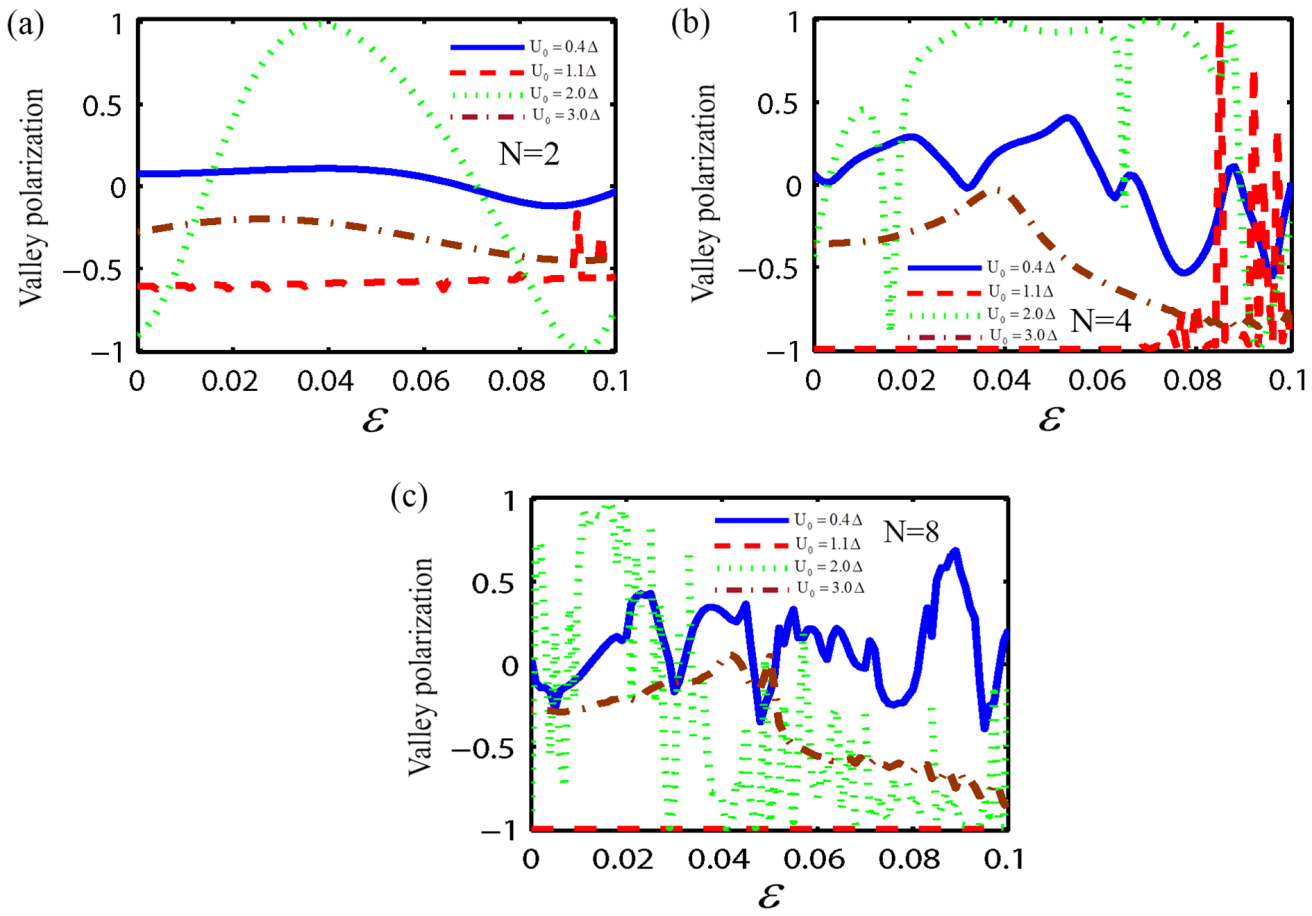
Dependence on the $\varepsilon$ of the valley spin polarization with several values of the height of the electrostatic potential barrier, for fixed $E = 1.5\Delta$, $\lambda_{R} = 50$ meV, $k_{F0} b = 5$ and for (**a**) two barrier, (**b**) four barriers, and (**c**) eight barriers structure.

In the following, we consider the effect of both the strain and the RSOC on the spin and valley dependent conductance and polarization through the monolayer MoS_2_ superlattice, as shown in Fig. 1. Here, we fix the parameters as; $E = 1.5\Delta$, $U_{0} = 3.5\Delta$, $\lambda_{R} = 50$ meV, the normalized barrier and the normal region width as $$k_{F0} b = 5\begin{array}{*{20}c} {(k_{F0} = E/\hbar v_{F} )} \\ \end{array}$$ and $k_{F0} w = 3$, respectively. First, we investigate the valley and spin-dependent conductance as a function of the strain strength ($\varepsilon$) with a different number of electrostatic potential barriers. As shown in Fig. 2, the conductance for the valley K_1_ depends on the spin degree of freedom and the number of the electrostatic barriers. Also for $N > 2$, the conductance without and with the spin-flip, shows an oscillatory behavior with respect to $\varepsilon$. Due to the strain and the normal regions' interface, more resonant peaks appear in the conductance by increasing the number of barriers in the superlattice. It is evident from Fig. 2 that the $G_{{ \uparrow \uparrow k_{1} }}^{{}} /G_{0}$ and $G_{{ \downarrow \uparrow k_{1} }} /G_{0}$ decrease by increasing the strain strength. For a large strain and when number of superlattice barriers is big enough ($N \ge 4$), spin-dependent conductance tends to zero. This is due to the evanescent states in the strain region. According to Fig. 2a, it is clear that for $\varepsilon \ge 0.08$, the value of $G_{{ \uparrow \uparrow K_{1} }}^{{}} /G_{0} = 0$ and $G_{{ \downarrow \uparrow K_{1} }}^{{}} /G_{0} \ne 0$. In other words, electrons could transmit through the monolayer MoS_2_ superlattice only with spin-flip, In this case the spin state of outgoing electrons were inverted by using monolayer MoS_2_ superlattice. So, the monolayer MoS_2_ superlattice acts as a spin inverter. The conductance for the valley K_2_ is presented in Fig. 3. It is observed that for $\varepsilon > 0.04$ the spin-dependent conductance, without and with spin-flip, in a monolayer MoS_2_ superlattice has a zero value due to the evanescent waves. This leads to a gap in the spin-dependent conductance with respect to the strain strength. Furthermore, when $\varepsilon < 0.04$, the conductance depends on the spin orientation of the electrons, and for $N > 2$, $G_{{ \uparrow \uparrow K_{2} }}^{{}} /G_{0}$ and $G_{{ \downarrow \uparrow K_{2} }}^{{}} /G_{0}$ shows an oscillatory behavior with $\varepsilon ,$ due to the propagating states in the strain region. According to Figs. 2 and 3, in a monolayer MoS_2_ superlattice, the conductance depends on both the spin and valley degrees of freedom. Thus, the spin and valley dependent conductance in a monolayer MoS_2_ superlattice can easily be adjusted by the strain. The other motivation of this paper is the calculation of the valley and spin polarization in a monolayer MoS_2_ superlattice under strain and RSOC. For this purpose, we first plot the spin polarization versus the strain strength, with a different number of barriers and several values of the height of the electrostatic potential barrier, in Fig. 4. According to Fig. 4, at first, by increasing the potential barrier's height, the spin polarization rises and reaches a maximum value. With the further increment of the electrostatic barrier height, the sign of the spin polarization is changed. In other words, the spin polarization shows great sensitivity to the number of electrostatic barriers and the barrier height, also the magnitude and sign of the spin polarization can be effectively manipulated by adjusting the number of barriers, the height of the electrostatic barrier, and the strain. The valley polarization is plotted in Fig. 5 as a function of the strain strength with a different number of barriers and several barrier height values. As shown in Fig. 5, the valley polarization has an oscillating evolution with the strain. Also, due to the oscillatory behavior of the conductance in terms of the barrier's height, the spin and valley dependent polarization will also show such behavior as a function of the barrier's height. Furthermore, it is seen from Fig. 5 that the valley polarization oscillates from -1 to 1 as $\varepsilon$ varies. By increasing the number of barriers in the MoS_2_ more resonant peaks appear in the valley and spin dependent polarization superlattice, due to the strain and the normal regions' interface. Consequently, the magnitude and the direction of the valley polarization can be tuned by the strain in a MoS_2_ superlattice. A fully valley polarized current occurs when the value of valley polarization is at its maximum values (1 or − 1). In this case, only the carriers from one valley (K or K′) will contribute to the conduction. Similar to the spin polarization, the number of the barriers and the barrier height are essential parameter in controlling the valley dependent transport properties in a monolayer MoS_2_ superlattice. These results imply that the MoS_2_ superlattice are excellent candidates for future straintronic and spintronics applications.

## Conclusion

In summary, we have investigated the spin and valley-dependent transport properties in a monolayer MoS_2_ superlattice under uniaxial strain and RSOC. We found that the strain has a significant effect on the spin and valley dependent conductance, without and with the spin-flip. Furthermore, we showed that the valley and spin dependent conductance have a gap regarding the strain, which allows the valley and spin conductance to have an on/off switching effect. More importantly, we produced both full spin and valley polarized current in a monolayer MoS_2_ superlattice under an armchair strain. Meanwhile, the magnitude and the direction of the valley and spin polarization can be tuned by strain, the number of electrostatic barriers, and barrier height. Our calculations indicated that the transport properties in a MoS_2_ superlattice under RSOC could be efficiently controlled mechanically.
